# Snowflake: A deep learning-based human leukocyte antigen matching algorithm considering allele-specific surface accessibility

**DOI:** 10.3389/fimmu.2022.937587

**Published:** 2022-07-29

**Authors:** Matthias Niemann, Benedict M. Matern, Eric Spierings

**Affiliations:** ^1^ Research and Development, PIRCHE AG, Berlin, Germany; ^2^ Center for Translational Immunology, University Medical Center, Utrecht, Netherlands; ^3^ Central Diagnostic Laboratory, University Medical Center, Utrecht, Netherlands

**Keywords:** HLA, epitope, epitope matching, antibodies, deep-learning, neural network, 3D-structures, structure prediction

## Abstract

Histocompatibility in solid-organ transplantation has a strong impact on long-term graft survival. Although recent advances in matching of both B-cell epitopes and T-cell epitopes have improved understanding of allorecognition, the immunogenic determinants are still not fully understood. We hypothesized that HLA solvent accessibility is allele-specific, thus supporting refinement of HLA B-cell epitope prediction. We developed a computational pipeline named Snowflake to calculate solvent accessibility of HLA Class I proteins for deposited HLA crystal structures, supplemented by constructed HLA structures through the AlphaFold protein folding predictor and peptide binding predictions of the APE-Gen docking framework. This dataset trained a four-layer long short-term memory bidirectional recurrent neural network, which in turn inferred solvent accessibility of all known HLA Class I proteins. We extracted 676 HLA Class-I experimental structures from the Protein Data Bank and supplemented it by 37 Class-I alleles for which structures were predicted. For each of the predicted structures, 10 known binding peptides as reported by the Immune Epitope DataBase were rendered into the binding groove. Although HLA Class I proteins predominantly are folded similarly, we found higher variation in root mean square difference of solvent accessibility between experimental structures of different HLAs compared to structures with identical amino acid sequence, suggesting HLA’s solvent accessible surface is protein specific. Hence, residues may be surface-accessible on e.g. HLA-A*02:01, but not on HLA-A*01:01. Mapping these data to antibody-verified epitopes as defined by the HLA Epitope Registry reveals patterns of (1) consistently accessible residues, (2) only subsets of an epitope’s residues being consistently accessible and (3) varying surface accessibility of residues of epitopes. Our data suggest B-cell epitope definitions can be refined by considering allele-specific solvent-accessibility, rather than aggregating HLA protein surface maps by HLA class or locus. To support studies on epitope analyses in organ transplantation, the calculation of donor-allele-specific solvent-accessible amino acid mismatches was implemented as a cloud-based web service.

## Introduction

Histocompatibility of patients and organ donors has been shown to correlate with long-term graft survival in organ transplantation (1). The formation and/or presence of antibodies towards the mismatched donor HLA plays an important role in that. Antibody recognition of donor HLA is dependent on the patient’s immune reactivity towards determinants on these mismatched donor HLA. The determinants that specifically interact with these antibodies have been designated as the antibody epitopes. The amino acid composition and tertiary structure of these donor epitopes may differ from the epitopes formed by recipients’ HLA proteins, allowing B-cell receptors to distinguish self epitopes from non-self epitopes, allowing for effective immune responses (2).

Several algorithms have been developed to convert HLA mismatches into B-cell epitope mismatches between two individuals. The HLAMatchmaker algorithm was the first HLA-centric algorithm to define antibody-accessibility based on a limited set of experimental crystal structures. The HLAMatchmaker algorithm groups amino-acid positions and their corresponding configurations for defining the so-called Eplets (3). The EPRegistry database has subsequently been developed as a central repository listing these Eplets (4, 5). By defining residue-specific physicochemical properties, potential explanations for epitope-specific antibody affinity were provided (6). The polymorphism of the HLA-gene region, compounded by pairing of two individuals in a transplantation setting, however, yields such a large number of combinations of HLA epitopes, that verifying each mismatched epitope indistinguishably by antibody reaction patterns is challenging if possible at all (7). Moreover, it has been suggested that basically every amino-acid mismatch is capable of triggering an immune response (8, 9). The HLA-EMMA algorithm exploits amino-acid mismatching, by only considering polymorphic amino-acid residues at solvent-accessible positions based on reported experimental structures of various HLA loci as potential B-cell receptor targets (10).

There are less than 100 distinct HLA Class I and less than 50 distinct HLA Class II crystal structures deposited in the RCSB Protein Data Bank (PDB, www.rcsb.org) (11), while more than 13,600 classical HLA Class I and more than 5,600 HLA Class II protein variants have been reported by the IPD-IMGT/HLA database [version 3.47 (12)]. Given this imbalance and the complex and time-consuming process of X-ray crystallography, it is not feasible to generate structure data for every HLA protein in order to identify surface residues. Thus, neural network predictors have been suggested to predict arbitrary proteins’ surfaces (13, 14). Refining and integrating aforementioned concepts, we developed a deep-learning prediction pipeline for solvent accessibility defining allele-specific surface epitopes. Our surface prediction is trained on experimental HLA structures of the PDB, which were supplemented by HLA structures predicted by DeepMind’s recent AlphaFold protein folding predictor (15–17). Due to the structural differences between HLA Class I and Class II proteins and the different distribution of available experimental structures, our prediction pipeline’s implementation needs specific optimization based upon the respective HLA classes. Herein, we present the results for our HLA Class I-specific pipeline. Based on our surface predictor, we implemented a matching algorithm as a web service allowing for real-time application in transplantation diagnostics. The full stack matching algorithm, named Snowflake, is available online (www.pirche.com), featuring machine-readable batch matching and a human-readable detail browser.

## Materials and methods

### Extraction of experimental structures from PDB

HLA protein structures were extracted from the PDB using the full text search term “hla”. The found structures were filtered by structures comprising chains “A”, “B” and “C”, whereas chain B’s amino-acid sequence was expected to match beta-2-microglobulin (UniprotKB entry P61769). The supposed alpha chains were aligned to the IPD/IMGT-HLA database (12) (version 3.46.0) using BLAST (18) *via* the Biopython library (19). Structures were only considered further if a BLAST score of 1300 was exceeded. HLA-allele names and identifiers were assigned to the protein structures based on the maximum number of identical amino acid configurations and lowest HLA identifier in case of a tie.

### Structural prediction of non-crystallized HLA proteins

Given the large number of known HLA genes and the complexity of generating structural data experimentally, the AlphaFold (15–17) (DeepMind Technologies Limited, London, UK) protein structure inference pipeline (v2.0) was applied to supplement the PDB data. As the public AlphaFold Database does not cover HLA variants, the AlphaFold software was installed on an Amazon Web Services Elastic Cloud Compute server (Amazon Web Services Inc., Seattle, US) to run *de novo* calculations. Generating HLA-protein 3D structures *via* AlphaFold is a heavy computational task. Consequently, only HLA proteins were selected for structure prediction when their serologic and antigenic groups were not already covered by structures from the PDB. For visualization purposes, structures were superimposed as described by Golub et al. (20) *via* the Biopython library (19).

### Render peptide in predicted structures’ binding groove

The inferred structures comprise the proteins’ respective alpha chain and the beta-2-microglobulin, but lack a bound peptide. The presence of such a peptide alters the solvent accessibility for residues involved in the peptide-binding domain. Despite AlphaFolds high prediction accuracy and capability to infer multimeric structures, it is not designed to predict binding of antibodies (17). To supplement the predicted structures with peptides, the Immune Epitope Database (IEDB, www.iedb.org) (21) was screened for nonameric peptides being reported as binders to the respective HLA protein. For predicted alleles without reported binders, the IEDB-reported peptides of the allele with the highest amino acid identity in the extracellular domains were considered as binders. For each allele, a subset of ten peptides was extracted and each peptide’s position in the HLA binding groove was predicted by the Anchored Peptide-MHC Ensemble Generator (APE-Gen) (22) to account for peptide-specific changes of accessibility.

### Structure comparison

**Figure 1:**
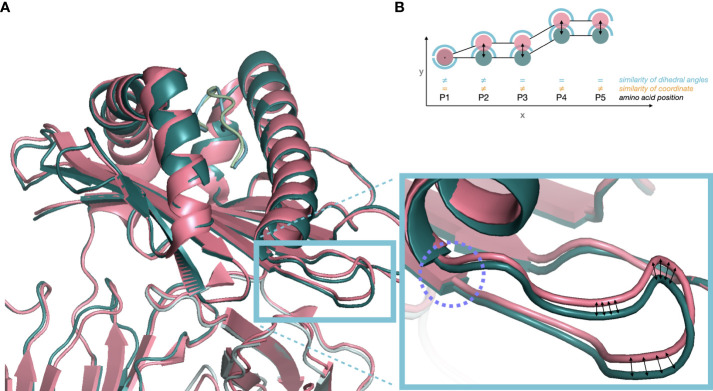
**(A)** Cartoon plot of two independent crystal structures of HLA-A*02:614 in overlay. In pink structure 3MRE (PDB DOI: 10.2210/pdb3MRE/pdb) and in green structure 3GSR (PDB DOI: 10.2210/pdb3GSR/pdb). Superimposition was based on atoms of identical amino acid residues. The blue box indicates small changes in dihedral angles (purple circle) propagating into large Euclidean distances in the following loop structure. **(B)** Euclidean distance between atom positions (orange) may overestimate the structural difference (P3-P5) due to distance-propagation of angular difference at P1/P2 (blue) or suboptimal global superimposition. However, dihedral angles at P3-P5 are identical, only considering P1/P2 as structurally different.


Comparing experimental protein structures by calculating the Euclidean distance of the respective residues’ coordinates is sensitive to local differences that propagate through the whole tertiary structure. The relatively small changes in dihedral angles in the backbone may twist the entire molecule, even though large functional units remain identically shaped. Increased Euclidean distances of all subsequent positions will thus be overrepresented. Furthermore, comparing Euclidean distances requires structures to be superimposed. Although axes units are identical, the experimental structures deposited in the PDB have varying coordinate centers and axes orientations, requiring prior computational superimposition of the structures, which may introduce additional bias. As a robust alternative, torsion angles of the amino acids’ backbones - characterized as dihedral angles phi (φ) and psi (ψ) - have been considered. The position-specific square of dihedral angle differences (SDA) and the overall root mean SDA (RMSDA) were calculated. Compared to evaluating Euclidean distances of atom pairs of identical amino acid sequence structures, RMSDA is superimposition-independent and local differences do not propagate through the structure (as reviewed by (23), **Figure 1**). Additionally, the position-specific square of solvent accessible surface area differences (SSA) and the overall root mean SSA (RMSSA) were calculated as a functional distance metric. Predicted structures of the five most frequent proteins of experimental structures reported in the PDB were compared to estimate the prediction performance. However, these predicted structures were not used in the neural network training data set.

$$SDA_{i}={\left(\varphi_{a_{i}}-\varphi_{b_{i}}\right)}^{2}+{\left(\psi_{a_{i}}-\psi_{b_{i}}\right)}^{2}$$

$$RMSDA=\sqrt{\frac{1}{2n}\sum_{i=1}^{n}{\left(\varphi_{a_{i}}-\varphi_{b_{i}}\right)}^{2}+{\left(\psi_{a_{i}}-\psi_{b_{i}}\right)}^{2}}$$

$$SSA_{i}={\left(SA_{a_{i}}-SA_{b_{i}}\right)}^{2}$$

$$RMSSA=\sqrt{\frac{1}{n}\sum_{i=1}^{n}{\left(SA_{a_{i}}-SA_{b_{i}}\right)}^{2}}$$

### Calculation of protein-residue-specific surface area

For all experimental and inferred HLA-protein structures, each residues’ solvent accessible surface area was calculated considering the Shrake-Rupley algorithm (24), implemented by the Biotite library (25). Essentially, this algorithm is the numerical equivalent of rolling a marble (i.e. probe) over a structure (i.e. HLA protein) to identify which parts of the structure (i.e. residues) get in contact with the marble. The probe radius was set to 1.4 Å corresponding to the size of water molecules. Surface area computation of alpha chain residues considered the proteins’ alpha chain, beta-2-microglobulin and bound peptide simultaneously.

### Neural network design

The experimental and inferred HLA structures comprise only a fraction of known HLA Class I proteins. To create a database of surface accessible residues for all HLA Class I proteins, a long short-term memory bidirectional recurrent neural network (BRNN) (26) was implemented, that chained the HLA alpha chains’ amino-acid configurations as one-hot-encoded inputs and the residues’ surface areas as output. The network configuration stacked a bidirectional layer with rectified linear unit (ReLU) activation of 100 neurons, a second bidirectional layer with 64 ReLU neurons, a third bidirectional layer with 32 ReLU neurons and the output layer considering a linear activation function. Position-specific surface area was limited to 100, corresponding to the 85th percentile, in order to account for protruding residues with disproportionate surface area. Surface area was divided by 100 resulting in values ranging from 0 to 1. Network training used Adam optimization for efficient gradient descent (27). The model-generation and inference was implemented in the Python programming language (Python Software Foundation, version 3.5.1) using Keras (https://keras.io)/Tensorflow 2 (https://www.tensorflow.org).

### Comparison of calculated surface area with other methods

The performance of the applied surface prediction pipeline was evaluated by comparing the predicted surface accessibility of 72 IMGT-IPD/HLA-reported HLA Class I reference alleles (based on IMGT 3.46.0 (28),) with the predictions provided by NetSurfP 2.0 (13) and PaleAle 4.0 (14). Relative solvent accessibility predicted by PaleAle was divided by 100 to match the other predictors’ outputs. The produced relative solvent accessibility scores were mean-centered. Position-specific squared error (SE) and mean squared errors (MSE) were calculated pairwise between the predictors and reference alleles. Amino acid sequence positions with large SE were visualized in PyMol 2.3.0. Amino acid positions with solvent accessibility scores exceeding the respective predictor’s mean solvent accessibility were considered accessible and inaccessible otherwise.

### Epitope matching based on surface-accessible amino acid residues

**Figure 2:**
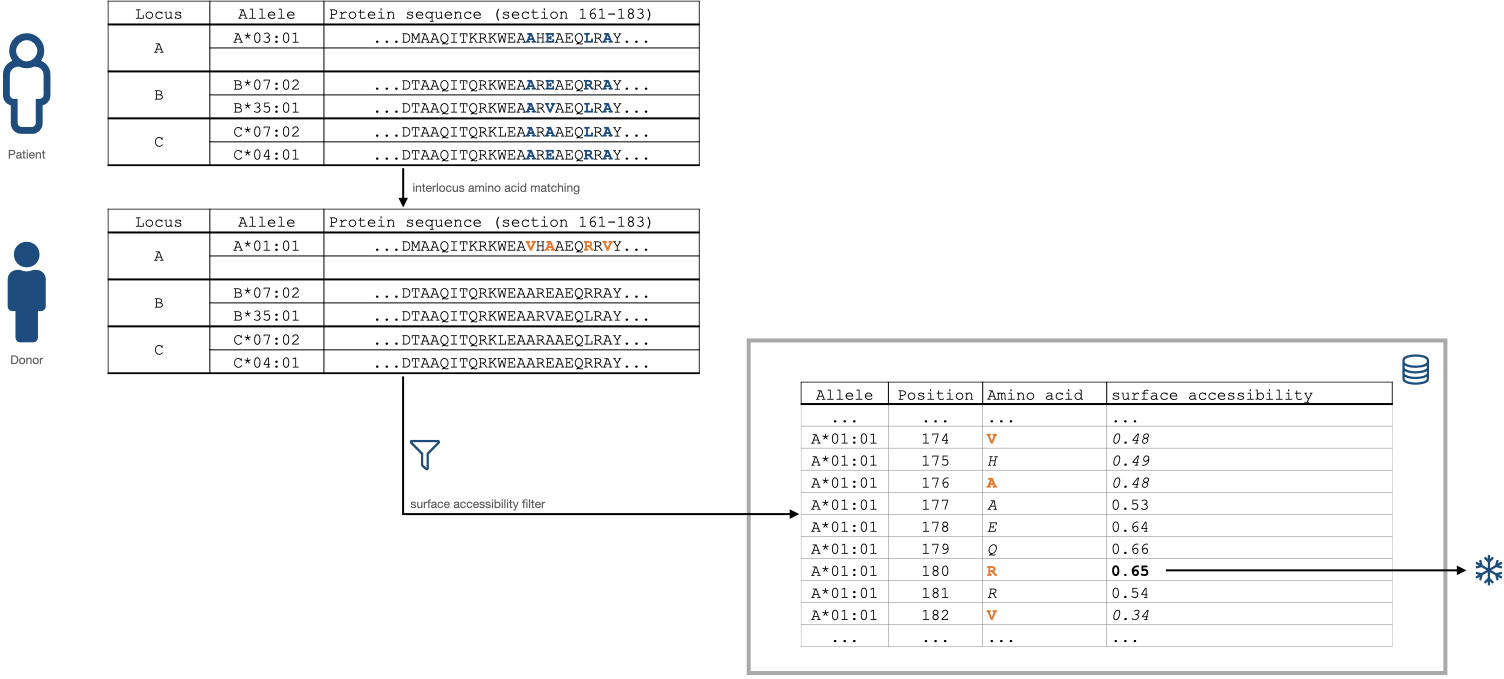
Snowflake HLA matching algorithm schematic. Donor amino-acid mismatches with a predicted threshold-exceeding allele- and position-specific solvent accessibility (0.00 to 1.00, 0.00 being inaccessible, 1.00 being exposed) increase the Snowflake score by one.

The proposed method considers donor amino-acid mismatches at allele-specific positions exceeding a surface accessibility threshold (i.e. are predicted to be surface-accessible) between two individuals as epitope mismatches (**Figure 2**). The sum of such amino acid mismatches is considered the Snowflake epitope mismatch score. The algorithm is parameterized in the surface accessibility threshold and the mode of reference loci: (1) intralocus matching considers only the same recipient locus, whereas (2) interlocus matching references all recipient’s HLA Class I proteins.

### Statistical analysis

All calculations were executed in R software (R 3.6.1, R Foundation for Statistical Computing, Vienna, Austria).

## Results

**Figure 3:**
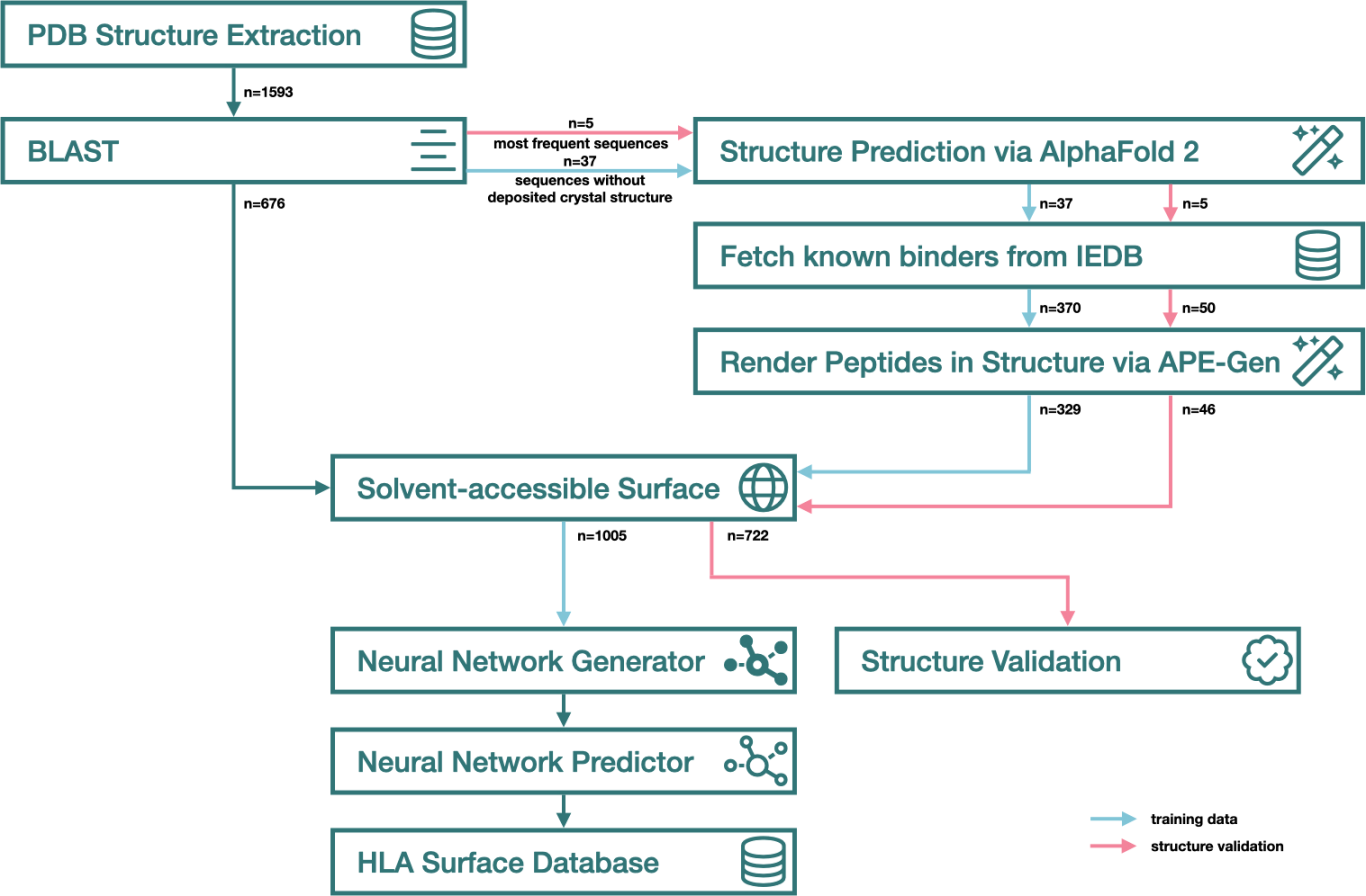
Snowflake surface accessibility prediction pipeline. Blue arrows represent training data, pink arrows represent validation data, green arrows represent data flow identical in both steps.

**Figure 4:**
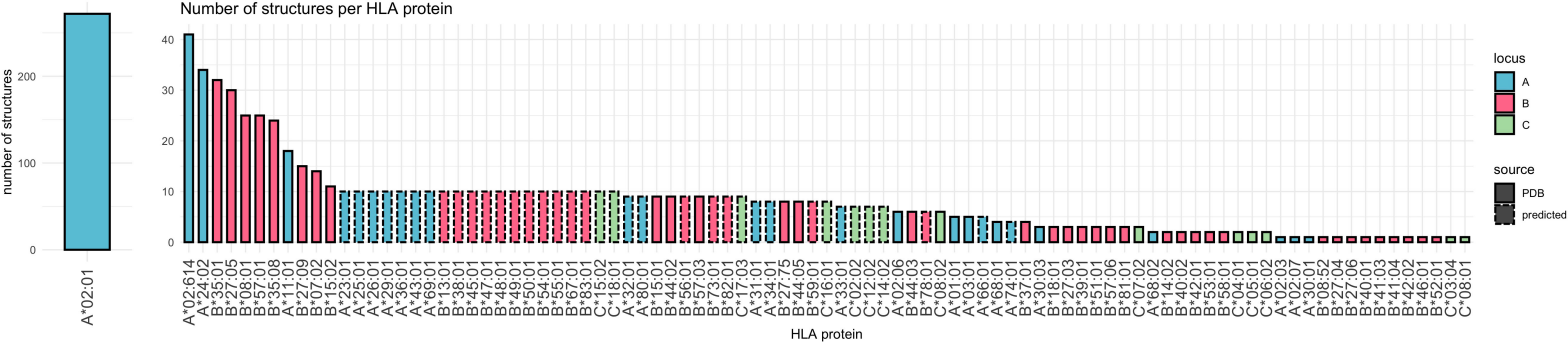
Bar chart indicating the distribution of alleles in experimental structures. The number of structures is encoded on the y axis. Color indicates locus, dashed border indicates structure prediction as outlined in the Materials and Methods section.

The PDB search yielded 1593 structures that were filtered down to 676 HLA Class-I experimental structures (**Figure 3**). The majority of experimental HLA Class I structures correspond to the A*02:01 (n=272, ~40%) (**Figure 4**). Based on the experimental structures’ distinct HLA proteins, an additional 37 Class-I alleles (14 HLA-A, 16 HLA-B, 7 HLA-C) were selected for structure prediction, covering all HLA Class I antigenic groups. Rendering epitopes into the binding grooves of 37 predicted HLA structures failed in 41 of 370 cases (median = 0, mean = 1.11) due to internal errors in APE-Gen where energy values exceed numeric limits. Distributions of position-specific solvent accessible surface area are provided in **Supplementary Figure 1**. Variance in surface area can be observed both for positions in interaction with the bound peptide (29), as well as residues outside the binding groove.

**Figure 5:**
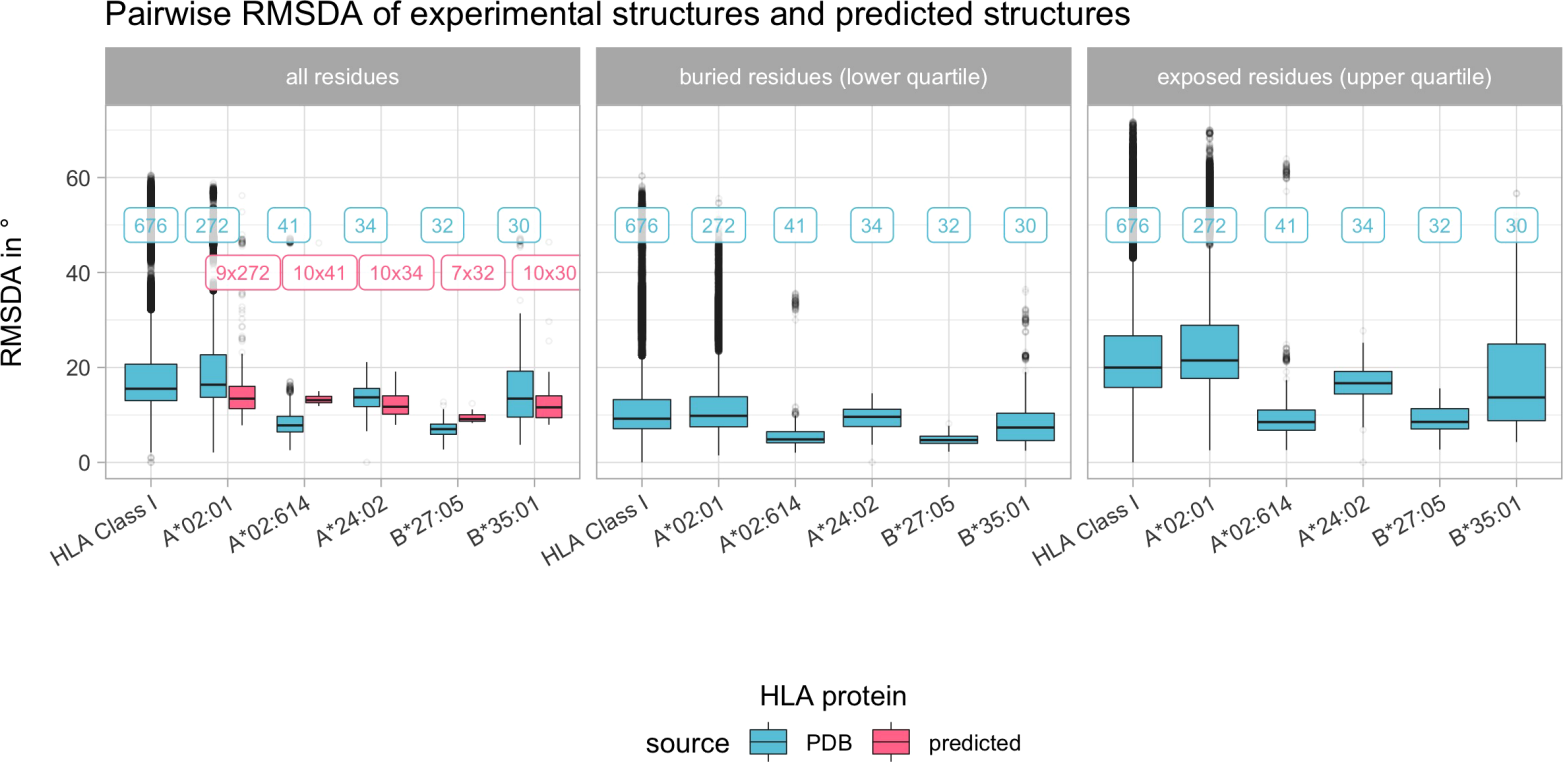
Boxplot indicating the pairwise root mean square dihedral angle differences (RMSDA) in degree across all experimental HLA Class I structures and pairwise across the most common HLA Class I structures. Color depicts the source of the structure data (blue indicates experimental structures from the PDB, pink indicates predicted structures as described in Materials and Methods). Horizontal panels consider separate distributions of all, only buried or only exposed residues. Boxplots depict the median (horizontal line), first to third quartile (box); the highest and lowest values within 1.5× IQR (whiskers) and outliers (circles), respectively. Labels refer to the number of structures being compared, with blue labels referring to experimental structures and pink labels referring to predicted structures being compared with experimental structures.

The distribution of pairwise RMSDA for the most common structures is similar compared to the overall RMSDA distribution considering structural variance across all HLA Class I proteins. Predicted structures’ RMSDA with their corresponding experimental structures is similar to the RMSDA deviation between experimental structures of the same allele (**Figure 5**). Certain regions of the HLA have higher SDA compared to rigid regions with low SDA (**Supplementary Figure 2**). Noteworthy, SDA does not correspond to levels of solvent accessibility (**Supplementary Figure 3**, Spearman’s rho = 0.135, p< 0.001).

**Figure 6:**
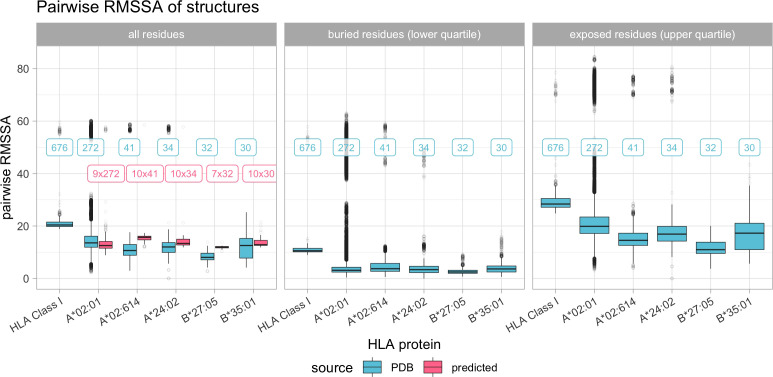
Boxplot indicating the pairwise root mean square surface area differences (RMSSA) across all experimental HLA Class I structures and pairwise across the most common HLA Class I structures. Color depicts the source of the structure data (blue indicates experimental structures from the PDB, pink indicates predicted structures as described in Materials and Methods). Horizontal panels consider separate distributions of all, only buried or only exposed residues. Boxplots depict the median (horizontal line), first to third quartile (box); the highest and lowest values within 1.5× IQR (whiskers) and outliers (circles), respectively. Labels refer to the number of structures being compared, with blue labels referring to experimental structures and pink labels referring to predicted structures being compared with experimental structures.

There is considerable variance in RMSSA observed within the various reported experimental structures of the same allele. The RMSSA across all HLA Class I experimental structures is however significantly higher compared to the allele-specific RMSSAs, indicating a higher variance between individual alleles (**Figure 6**). Also for the position-wise variance of surface area, certain regions of the HLA are subject to higher variance in SSA than others (**Supplementary Figure 4**).

### Compare surface prediction with reference tools

**Figure 7:**
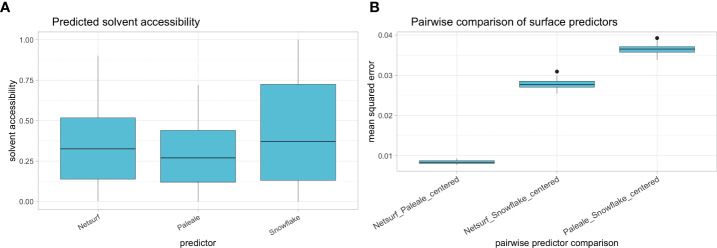
**(A)** Solvent accessibility distribution of reference alleles as predicted by NetSurfP (i.e. “rsa”), PaleAle (i.e. Relative Solvent Accessibility) and Snowflake. **(B)** Pairwise mean squared distance between centered predictions of NetSurfP and PaleAle, NetSurfP and Snowflake, and PaleAle and Snowflake. Boxplots depict the median (horizontal line), first to third quartile (box); the highest and lowest values within 1.5× IQR (whiskers) and outliers (circles), respectively.

After training the Snowflake surface predictor over 400 epochs, the loss function converged to ~0.02, so further training did not alter neural network weights. The average predicted relative solvent accessibility ranged lower for NetSurfP and PaleAle (i.e. reference predictors) compared to Snowflake (**Figure 7A**). After centering, the reference predictors produced similar predictions with low MSE across reference alleles (**Figure 7B**). Snowflake had considerably higher MSEs considering the reference predictors (**Figure 7B**).

**Figure 8:**
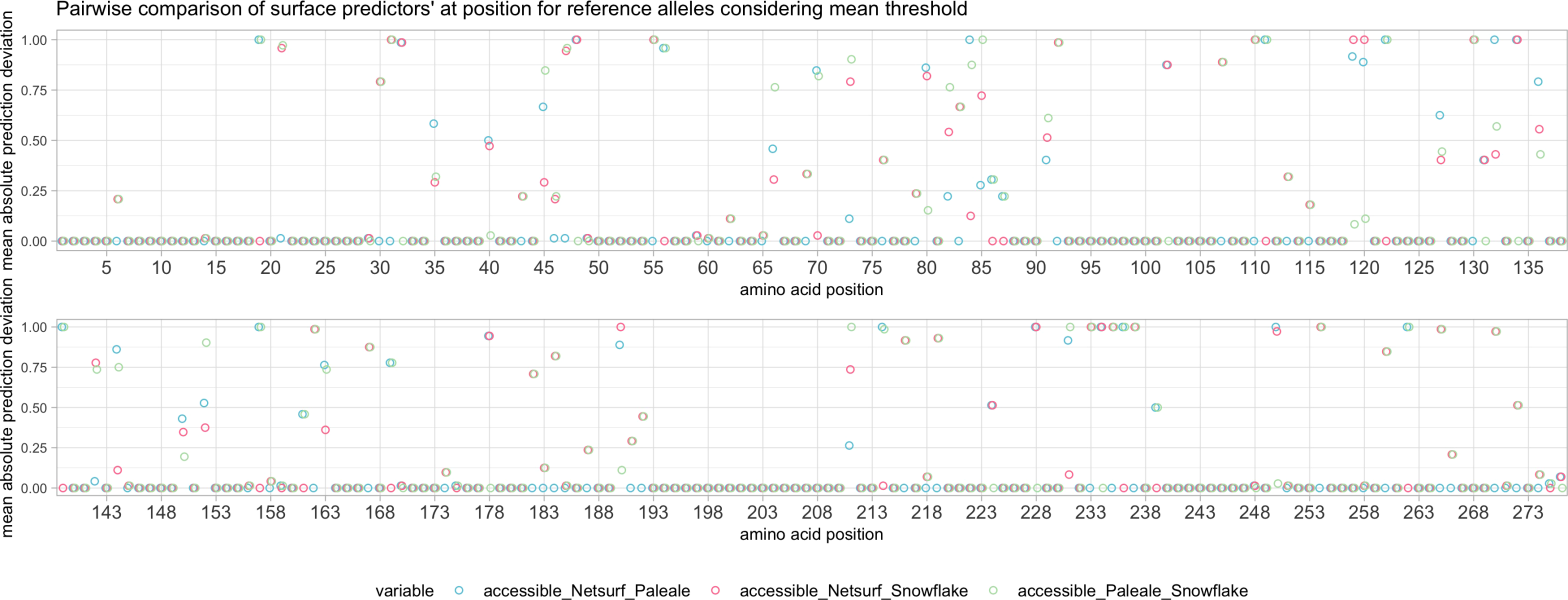
Pairwise comparison of predictions considering reference alleles with respective predictor’s mean as cutoff for surface accessibility.

The low MSE between NetSurfP and PaleAle was also observed in the low position-specific SE (**Supplementary Figure 5**) at the majority of amino acid positions. Position-specific SE for Snowflake was substantially higher in certain regions (**Supplementary Figure 5**). Classifying solvent accessibility by exceeding the respective predictor’s mean score yielded a considerable number of allele-dependent (e.g P3, P110, P190) and allele-independent (e.g. P31, P167 or P219) deviations between the Snowflake and the reference predictors (**Figure 8**). As an example, the solvent accessibility prediction and SE of A*02:01 have been provided in **Supplementary Figure 6**.

**Figure 9:**
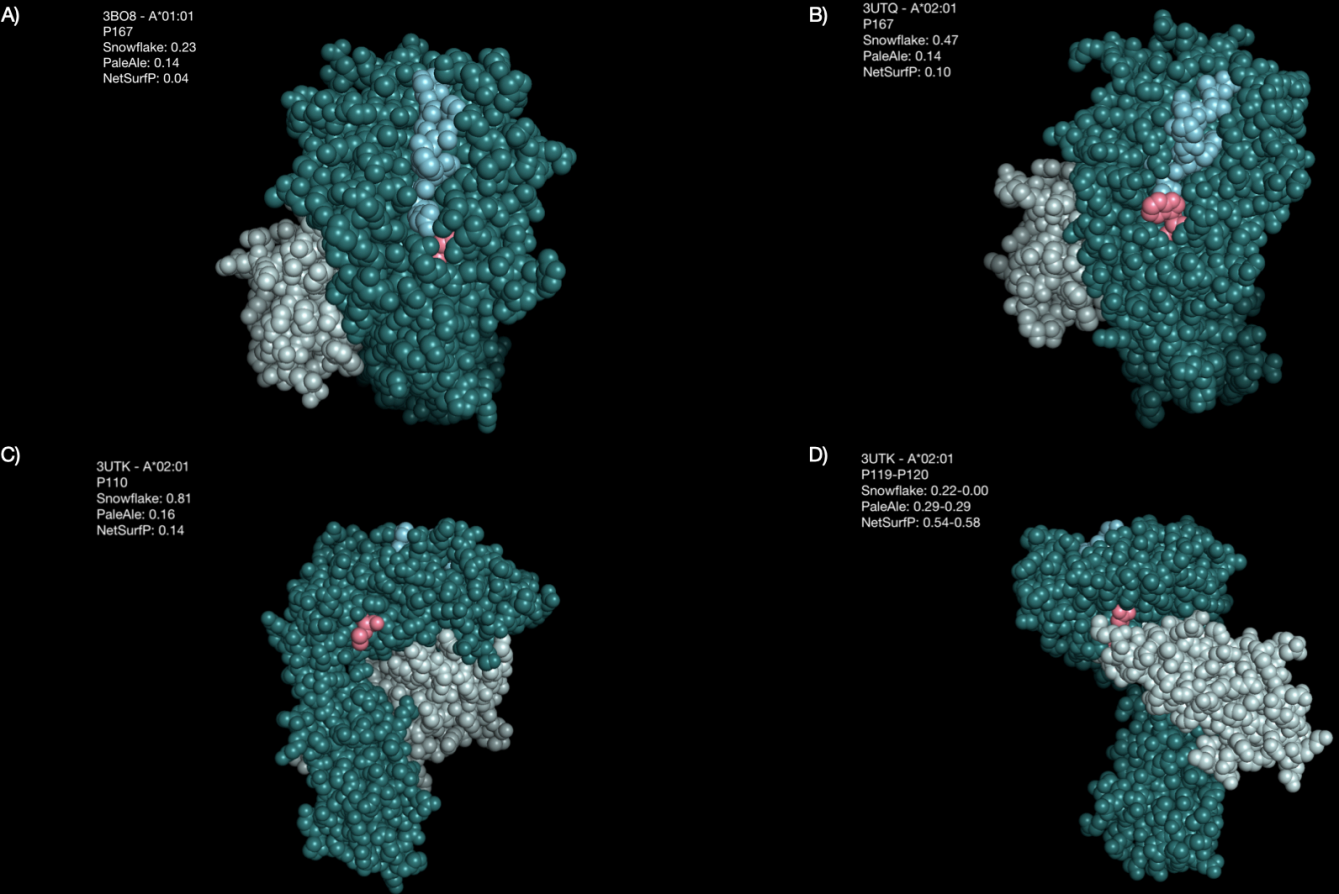
Highlighted amino acid positions of selected structures. The HLA alpha chain is colored in dark green, beta-2-microglobulin is colored in light green, the bound peptide is colored in blue, and the highlighted amino acid positions are colored pink. **(A)** Position P167 is predicted by Snowflake and the reference predictors to be buried in A*01:01. **(B)** The same position P167 in A*02:01 is predicted by Snowflake to be accessible, by the reference predictors again predicted to be buried. **(C)** The reference predictors estimate position P110 in A*02:01 to be buried, whilst Snowflake considers it accessible. **(D)** For A*02:01, positions P119 and P120 are contact points with the beta-2-microglobulin. Whilst Snowflake considers these positions not being accessible, NetSurfP considers them as accessible.

Generating visual representations of some of these characteristic positions indicates allele-specific differences in surface prediction (**Figure 9**). Amino acid position 167 of A*01:01 is not predicted to be solvent-accessible by Snowflake, PaleAle and NetSurfP. Despite PaleAle and NetSurfP predicting the same position also not being accessible in an A*02:01, Snowflake estimates P167 being accessible (**Figure 9B**). In the visual representation accessibility appears plausible. Notably, positions 166 and 167 of A*01:01 have been described by the Eplet 166DG. Although the corresponding amino acid positions of A*02:01 are both surface accessible, no corresponding Eplet 166EW is defined.

For position 110 of A*02:01 the predictors disagree on accessibility; PaleAle and NetSurfP negate solvent accessibility, whilst Snowflake affirm accessibility. By visual representation, accessibility of this position however appears plausible (**Figure 9C**).

**Figure 10:**
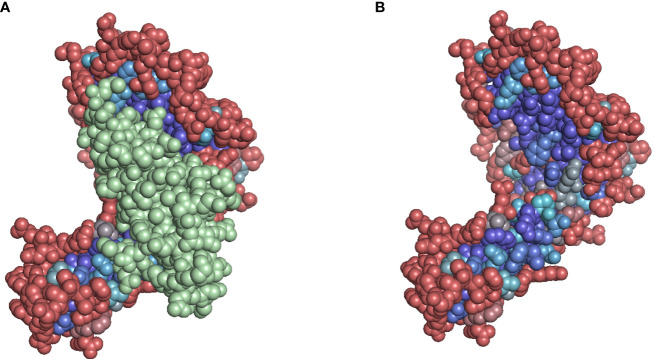
**(A)** Solvent accessibility-colored residues of A*02:01. Beta-2-microglobulin is colored in green. **(B)** Residues masked by the beta-2-microglobulin structure are predicted to be inaccessible. Color (gradient from red to purple to blue) based on Snowflake score, red tones indicating higher solvent accessibility, blue tones indicate lower solvent accessibility.

The positions 119 and 120 of A*02:01 appear to be masked by the beta-2-microglobulin structure (**Figure 9D**). Opposed to Snowflake and PaleAle both agreeing on the inaccessibility of these positions, NetSurfP predicts accessibility. Coloring the residues depending on their predicted solvent accessibility, shows the masking effect of the bound beta-2-microglobulin, rendering hidden amino acid positions, despite being potentially polymorphic, antibody-inaccessible (**Figure 10**).

**Figure 11:**
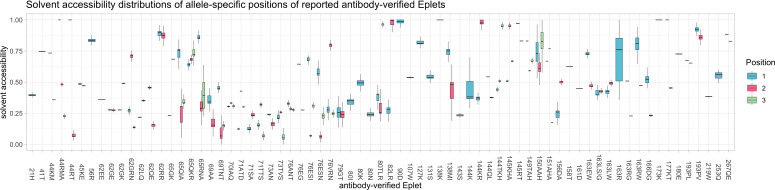
Distributions of solvent accessibility of respective antibody-verified Eplets positions. Color corresponds to the amino acid position within the Eplet. Boxplots depict the median (horizontal line), first to third quartile (box); the highest and lowest values within 1.5× IQR (whiskers), respectively.

HLA Class I antibody-verified eplets are defined as one to three amino acids. At each of respective positions, distinct distributions of solvent accessibility were observed (**Figure 11**), leading to different patterns of solvent accessibility: (i) Eplets with consistently high predicted solvent accessibility (e.g. 127K, 193PV), (ii) Eplets with a consistent pattern of solvent accessibility, where at least one amino acid position of the Eplet has high predicted solvent accessibility (e.g. 145KHA, 44RT), and (iii) Eplets with high variance of predicted solvent accessibility at one or more positions (e.g. 144K, 163R, 65RNA). Similar patterns were observed for non-antibody-verified Eplets (**Supplementary Figure 7**). Compared to antibody-verified Eplets, positions of non-antibody-verified Eplets were more often predicted to be inaccessible.

**Figure 12:**
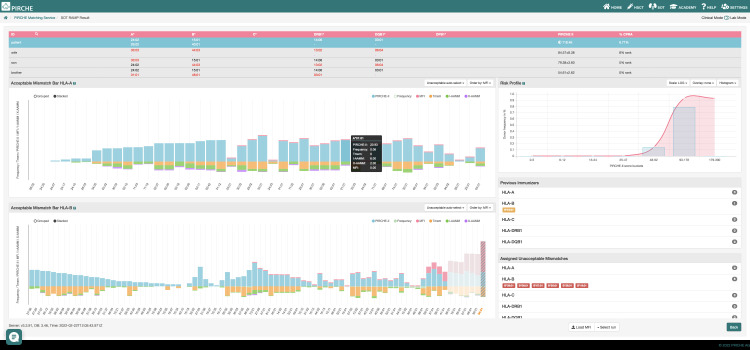
PIRCHE Risk and Acceptable Mismatch Profile including PIRCHE-II epitope scores (blue bars), mean fluorescence intensities of imported single antigen bead assays (pink bars), shared T-cell epitopes with previous immunizers (orange bars), allele frequencies (pale green bars), interlocus Snowflake scores (purple bars) and intralocus Snowflake scores (dark green bars).

Snowflake matching was integrated in the previously described PIRCHE Risk and Acceptable Mismatch Profile epitope matching suite (30). The PIRCHE web service allows semi-automated HLA typing data import (GL String, HML, CSV) and provides interfaces to major HLA typing kit vendors’ software suites to load HLA typing data quickly and error-free. Interactive bar charts provide interlocus and intralocus Snowflake scores along with PIRCHE scores, allele frequencies, predicted shared T cell epitopes and mean fluorescence intensities of single antigen bead assays (**Figure 12**).

## Discussion

A wide range of universal software tools characterizing potential B-cell epitopes has been described in the literature (e.g. ElliPro (31), NetSurfP (13), PaleAle (14), DiscoTope (32), BepiPred (33)). Specifically for transplant histocompatibility matching, these tools supported approaches of HLA B-cell epitope definition and matching. HLAMatchmaker (3) (www.epitopes.net) and EPRegistry (4, 5) (www.epregistry.com.br) define a manually curated database of antibody-accessible amino acid configurations (i.e. eplets) based on a limited number of experimental HLA structures. Verifying each of these eplets by antibodies and estimating their immunogenicity however remains challenging (7, 34). The EMS-3D model considers a multidimensional score of electrostatic dissimilarity between donor and recipient HLA, which may be an additional metric to characterize the HLA surface (6).

In the present study, we describe the Snowflake algorithm, a newly developed deep learning-based HLA-matching algorithm that considers allele-specific surface accessibility. Snowflake applies a strategy comparable to the one used by the pHLA3D database (phla3d.com.br) (35). Both approaches complement the experimental structures of the PDB by *in silico* predicted structures of pHLAs using a homology modeling pipeline approach in order to spatially locate amino acid positions in HLA proteins without available structures. Snowflake applies AlphaFold for structure prediction as it appears to outperform the “satisfaction of spatial restraints”-approach of MODELLER, which was used to construct the pHLA3D database (15). As Snowflake considers multiple binding peptides during training, peptide-specific bias in solvent accessibility of binding groove residues is taken into account. Comparing the Snowflake-predicted surface accessibility with the calculated solvent-accessible surface area of pHLA3D database structures confirms a concordance between the approaches for the majority of amino acid positions (**Supplementary Figure 8**). However, each structure has a number of amino acid positions with large deviation between pHLA3D and Snowflake (i.e. outliers) indicating differences in parts of the predicted structures. Considering protein flexibility and different optimization strategies, these differences may however be technical artifacts of the respective tool chain’s characteristics.

To incorporate simulated HLA structures into surface prediction, Snowflake implemented its own HLA surface predictor comparable to both NetSurfP 2.0 and PaleAle 4.0. Similar to Snowflake, these predictors implement a deep learning neural network architecture using BRNN to predict (amongst other characteristics) relative surface accessibility. Both networks have been trained with the experimental structures reported in the PDB (13, 14). Limiting on solvent accessibility as predicted output, Snowflake’s deep learning model has been trained exclusively on HLA Class I structures, tailoring its prediction capabilities specifically to HLA Class I proteins. The observed lower numerical distance between predictions of NetSurfP and PaleAle compared to Snowflake and these predictors (**Figure 7**) may be caused by a different strategy in preprocessing calculated surface area. Considering predicted structures as additional training data is an advantage over NetSurfP and PaleAle, as it incorporates domain-specific knowledge on protein folding into solvent accessibility prediction. It must be considered that learning-based surface prediction is obsolete if the predicted structures have been generated for all known HLA alleles. Proving the predictors’ reliability and accuracy requires more experimental structures of HLA proteins that have not been analyzed so far. Given the complexity of X-ray crystallographic experiments, this remains challenging.

Although HLA Class I experimental structures showed little overall structural variance in dihedral angles between alleles (**Figure 5**), solvent accessibility varied across different alleles, indicating solvent accessibility to be allele-specific (**Figure 6**). Visual representations of characteristic positions support this hypothesis (**Figure 9**). Consequently, amino acid matching approaches may be improved by considering solvent accessibility of the respective amino acid of the specific allele, providing a consistent explanation for epitopes’ directionality.

Despite strong numeric correlation between Eplets and Snowflake, some difference in epitope definition may arise. The example in **Figure 9** shows strong accessibility for positions 166 (glutamic acid) and 167 (tryptophan) of A*02:01, yet there is no Eplet 166EW defined. As described by (3), the interlocus comparison with HLA-B and -C typically shows matched monomorphic configurations at these positions. However, in the meantime there are HLA-B and -C surface-expressible alleles described, which do not share the EW configuration at these positions (data not shown). Consequently, there may be scenarios where a common hypothetical 166EW is not found in the recipient’s self-Eplets, potentially provoking an immune response. Similar examples are 253Q without a corresponding common 253E or 131S without a corresponding common 131R. Although these situations are likely rare, this indicates potential gaps in Eplet definitions. Furthermore, verifying Eplets with low population frequency by antibody reaction patterns remains challenging. The herein presented definition of epitope mismatches provides a consistent allele-specific process.

The position-specific solvent accessibility at amino acid positions encoding for antibody-verified Eplets (**Figure 11**, **Supplementary Figure 7**) varies for some of the reported Eplets. This variation suggests that the three-dimensional structure of such Eplets is dependent on the complete structure of the allele they are occurring in. This dependency raises the question, if antibody reactions against these Eplets are equally pronounced for alleles with lower accessibility compared to alleles with higher accessibility of the corresponding amino acid positions. Accessibility of non-antibody-verified Eplets generally appeared to be comparatively lower. The lower accessibility may be the cause for lack of antibody verification in the first place, and supports the hypothesis that non-antibody-verifiable internal Eplets act as proxies by impacting the protein surface from within.

HLA epitope-matching algorithms based upon solvent-accessible amino-acid mismatches are valuable tools in antibody-based HLA-mismatch risk classifications. The recently described HLA-EMMA (hla-emma.com) algorithm considers these solvent-accessible amino acid mismatches as potential B-cell receptor targets by defining solvent-accessible amino acid positions per HLA locus. The HLA-EMMA algorithm considers an amino acid position as solvent-accessible, if PaleAle- or NetSurfP-solvent accessibility scores exceed a certain threshold in at least one of the considered alleles of a locus (10). This approach may, however, assign mismatched amino acids of two alleles as epitope mismatch even though the amino acids within the respective alleles aren’t surface-accessible (**Figures 8**, **9A, B**). Such misclassifications can result from the fact that accessibility of the involved position was determined by a structural alignment to a different allele. Consequently, by using an allele-specific approach, Snowflake’s allele-specific solvent accessibility of mismatched amino-acids may increase specificity.

To confirm the impact of allele-specific solvent accessibility experimentally, binding analyses of HLA epitope-specific monoclonal antibodies to HLA as applied by Bezstarosti et al. may be informative (36). Following the hypothesis, antibodies against antibody-verified Eplets with a wide distribution of surface accessibility (e.g. 138MI or 163R, **Figure 11**) are expected to yield into a binding pattern proportional to the respective alleles’ Eplet-accessibility. Linked mismatched amino acid residues may however interfere with the complete antibody footprint, which has to be considered as a potential confounder of such experiments.

To fully evaluate the predictions of Snowflake, it is necessary to consider potential limitations. Our solvent accessibility prediction considered structural data generated by a protein folding predictor. Such an approach is essential due to limited and biased data of experimental HLA structures. Despite advances in the field of protein folding predictors (37), it must be noted protein folding is one of the most challenging problems in bioinformatics with predictions still lacking precision (38). Our data indicate considerable structural variance between experimental structures of the same allele, which may be explained by the varying specific experimental setups on the one part and by the flexibility of HLA molecules on the other (39, 40). Though predicted solvent accessibility varies to a greater extent across different alleles, protein flexibility and dynamics may still alter the protein surface *in vivo*.

Second, validation of AlphaFold-predicted structures did not account for structures being part of the training set and thus may overestimate the suitability of rendered structures for epitope analyses. Predicted positions of peptides in predicted HLA structures only considered nonameric peptides. The method may be further refined by including peptides of varying lengths depending on length distributions of reported binders, to evaluate the role of peptide length on solvent accessibility in the groove-surrounding region.

Training data for surface prediction is biased towards HLA-A*02:01 (**Figure 4**). Excluding structures based on sequence similarity as suggested previously (13, 14) would, however, significantly limits the training set. Considering the training data bias being not only sequence-specific but also position-specific, as positions have individual amino acid distributions across HLA alleles, training data aggregation at the allele level is insufficient. Lacking a robust aggregation option and accepting the potential bias, the complete data sets were used for training.

The applied surface area calculation considered a probe radius of 1.4 Å based on the size of water molecules. Given the size of HLA-binding antibodies and the interaction area between HLA and antibody (e.g. in PDB 6ID4 (41)), it may however be considered to restrict antibody accessibility to larger probe sizes or aggregate solvent accessibility with predicted ellipsoid protrusion.

Similar to all previously described HLA epitope matching algorithms, the Snowflake core algorithm also requires protein-level HLA typing data. By applying the previously described haplotype frequency-based multiple imputation (42), Snowflake scores can however be calculated with low- or intermediate resolution HLA typing data. To the best of our knowledge, Snowflake is the first HLA B cell epitope matching algorithm natively supporting low-resolution typing inputs. The Snowflake matching algorithm for batch processing or detailed matching is available at pirche.com.

In summary, we herein presented the Snowflake B-cell epitope matching algorithm, a deep-learning based software evaluating allele-specific surface-accessible amino acid mismatches applicable to all HLA Class I alleles. It refines protein surface-restricted amino acid matching in the context of transplantation and may support analyses on the immunogenicity and antigenicity of HLA B-cell epitopes. Clinical studies are warranted to indicate correlation between the Snowflake score and immunological events, such as development of donor-specific antibodies.

## Data availability statement

Publicly available datasets were analyzed in this study. This data can be found here: RCSB PDB: https://www.rcsb.org, Epitope Registry: https://www.epregistry.com.br, AlphaFold predictor: https://alphafold.ebi.ac.uk, Immune Epitope DataBase: https://www.iedb.org.

## Author contributions

MN and ES contributed to conception and design of the study. BM and MN extracted data. MN developed the prediction pipeline and wrote the first draft of the manuscript. All authors contributed to manuscript revision, read, and approved the submitted version.

## Funding

This study is supported by the German Federal Ministry for Economic Affairs and Climate Action (grant 01MJ21002B).

## Conflict of interest

MN works for PIRCHE AG, which develops and operates the PIRCHE web service. PIRCHE AG and UMC Utrecht have filed a patent application on the prediction of an alloimmune response against allele-specific solvent-accessible amino acid mismatches. MN and ES are listed as inventors on this patent.

The remaining author declare that the research was conducted in the absence of any commercial or financial relationships that could be construed as a potential conflict of interest.

## Publisher’s note

All claims expressed in this article are solely those of the authors and do not necessarily represent those of their affiliated organizations, or those of the publisher, the editors and the reviewers. Any product that may be evaluated in this article, or claim that may be made by its manufacturer, is not guaranteed or endorsed by the publisher.

## Abbreviations

APE-Gen, Anchored Peptide-MHC Ensemble Generator; BRNN, bidirectional recurrent eural network; IEDB, Immune Epitope DataBase; HLA, human leukocyte antigen; MSE, mean squared error; PDB, Protein Data Bank; ReLU, rectified linear unit; RMSDA, root mean square of dihedral angle differences; RMSSA, root mean square of solvent accessibility differences; SDA, square of dihedral angle difference; SE, squared error; SSA, square of solvent accessibility difference.
