# Supplementary material for: Snowflake: A deep learning-based human leukocyte antigen matching algorithm considering allele-specific surface accessibility
Source: Front Immunol. 2022 Jul 29;13:937587. doi: 10.3389/fimmu.2022.937587 (PMC9372366; doi:10.3389/fimmu.2022.937587)
Supplement: Supplementary file 1 [file DataSheet_1.docx]

# ***Supplementary Figures***


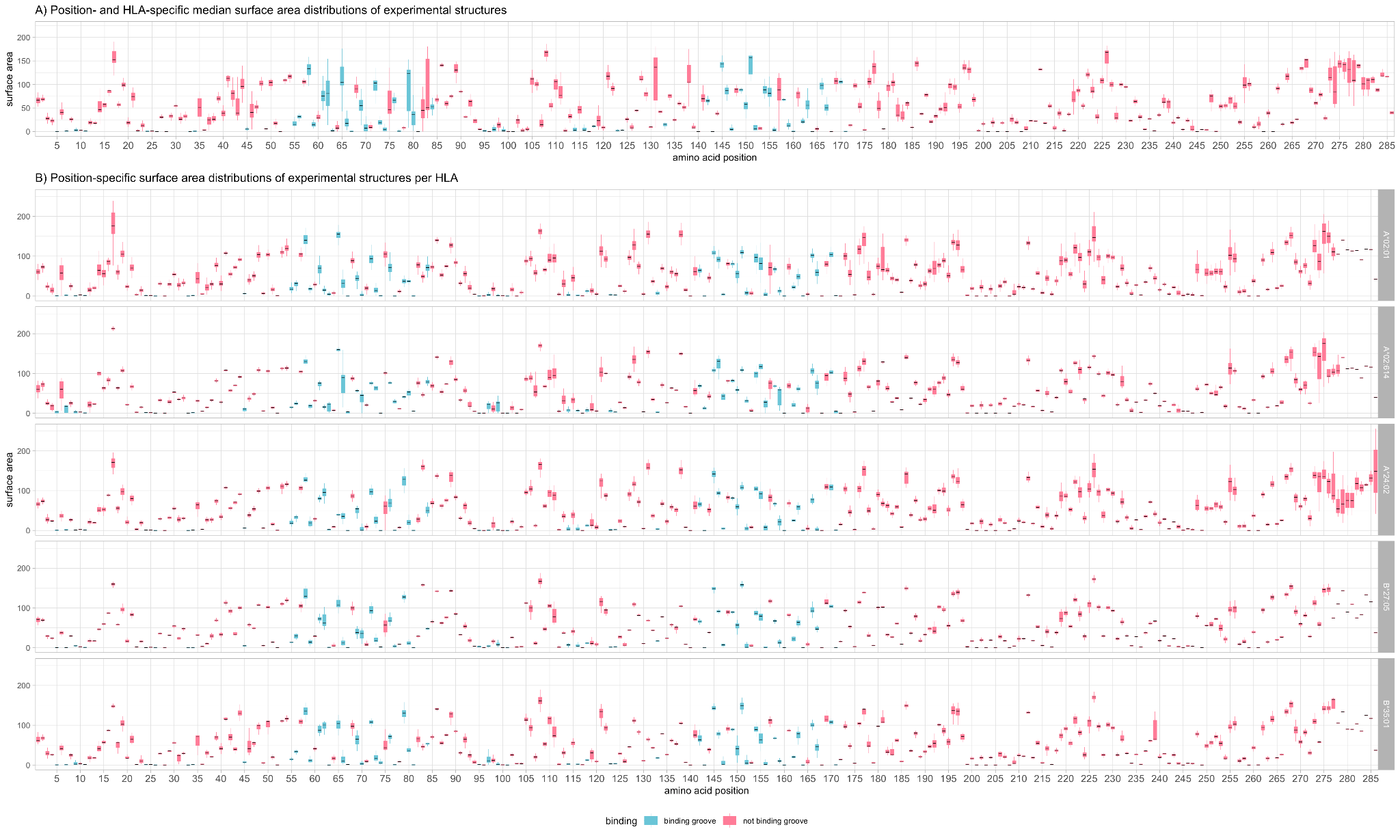


Supplementary Fig 1: Distribution of solvent accessible surface area aggregated (A) across all considered experimental structures or (B) considering the most frequent alleles deposited in the PDB. Residue positions are encoded on the x axis, surface area on the y axis. Color indicates the amino acid positions of the binding groove as defined by [[28]](https://www.zotero.org/google-docs/?VdbEI8). Boxplots depict the median (horizontal line), first to third quartile (box); the highest and lowest values within 1.5× IQR (whiskers), respectively.


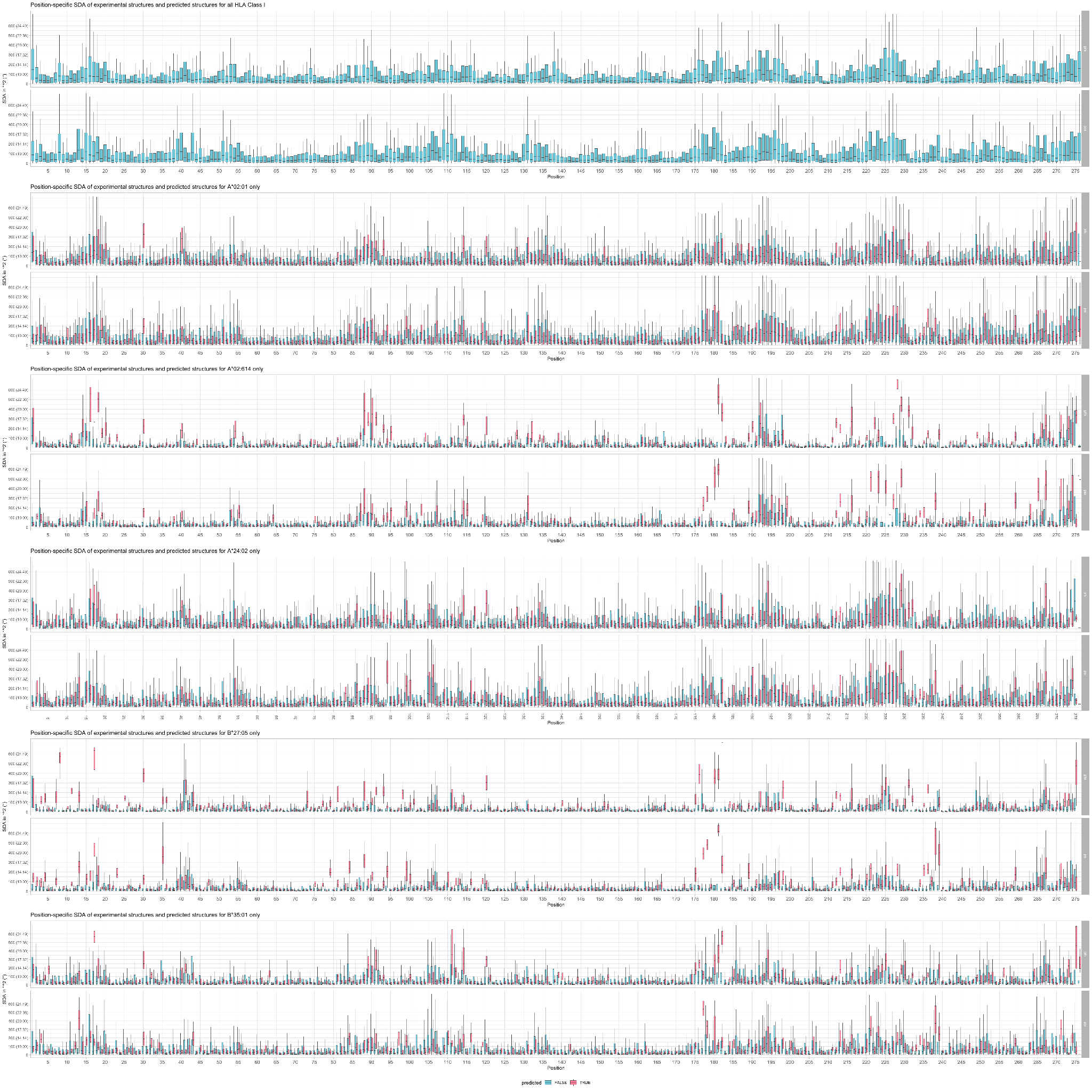


Supplementary Fig 2: Position-specific square dihedral angle difference (y axis) in phi/psi angles (paired rows). Residue positions are encoded on the x axis. Top rows indicating all experimental HLA Class I structures, following rows indicating the alleles with the most structural datasets in the PDB (A*02:01, A*24:01, B*27:05, B*35:01, A*02:614). Blue boxes depict pairwise SDA of experimental structures, pink boxes depict pairwise SDA of predicted structures with experimental structures. Boxplots depict the median (horizontal line), first to third quartile (box); the highest and lowest values within 1.5× IQR (whiskers), respectively.


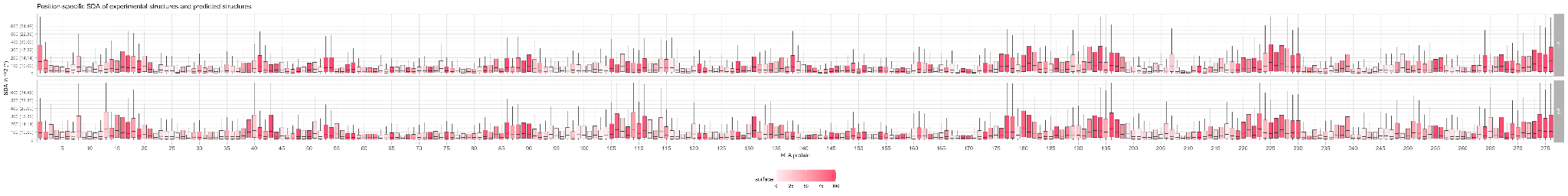


Supplementary Fig 3: Position-specific square dihedral angle difference (y axis) in phi/psi angles (rows) considering all experimental HLA Class I structures. Residue positions are encoded on the x axis. Color corresponds to the position’s median solvent accessibility with low SDA corresponding to white, high SDA corresponding to pink. Boxplots depict the median (horizontal line), first to third quartile (box); the highest and lowest values within 1.5× IQR (whiskers), respectively.


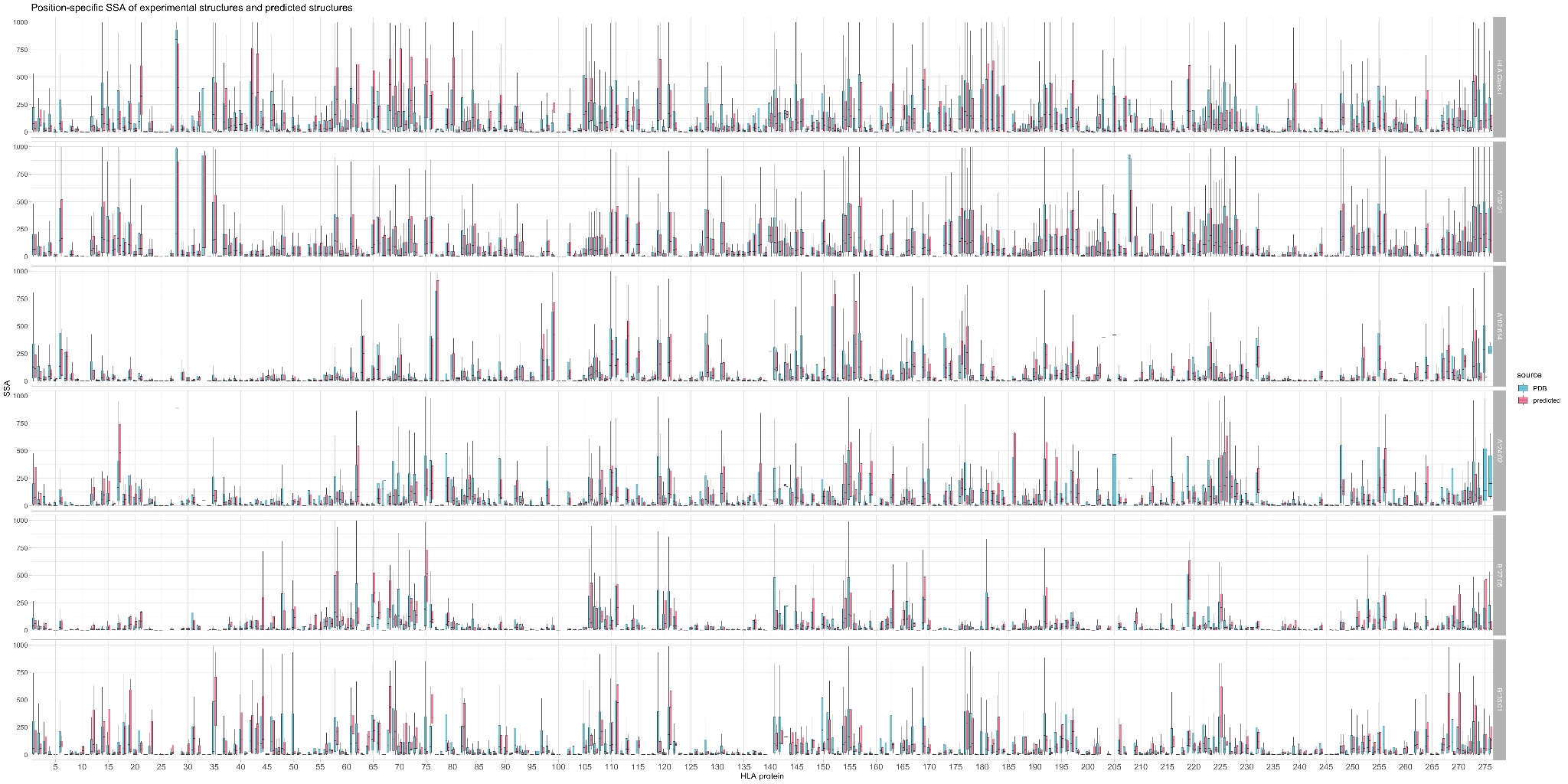


Supplementary Fig 4: Position-specific square surface area difference (y axis). Top row indicating all experimental HLA Class I structures, following rows indicating the alleles with the most structural datasets in the PDB (A*02:01, A*24:01, B*27:05, B*35:01, A*02:614). Residue positions are encoded on the x axis. Blue boxes depict pairwise SSA of experimental structures, pink boxes depict pairwise SDA with the predicted structure. Boxplots depict the median (horizontal line), first to third quartile (box); the highest and lowest values within 1.5× IQR (whiskers), respectively.


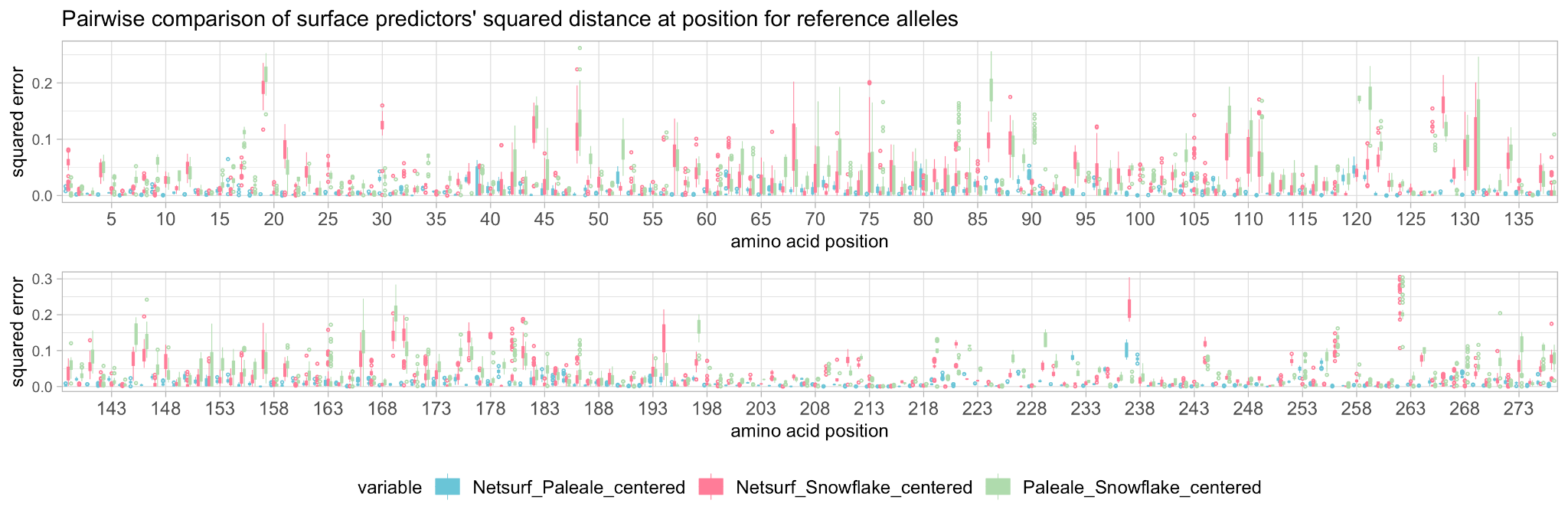


Supplementary Fig 5: Position-specific squared error of pairwise predictors’ solvent accessibility score (y axis) comparisons considering 72 reference alleles. Residue positions are encoded on the x axis. Blue boxes/circles: NetSurfP vs. PaleAle, pink boxes/circles: NetSurfP vs. Snowflake, green boxes/circles: PaleAle vs. Snowflake. Boxplots depict the median (horizontal line), first to third quartile (box); the highest and lowest values within 1.5× IQR (whiskers) and outliers (circles), respectively.


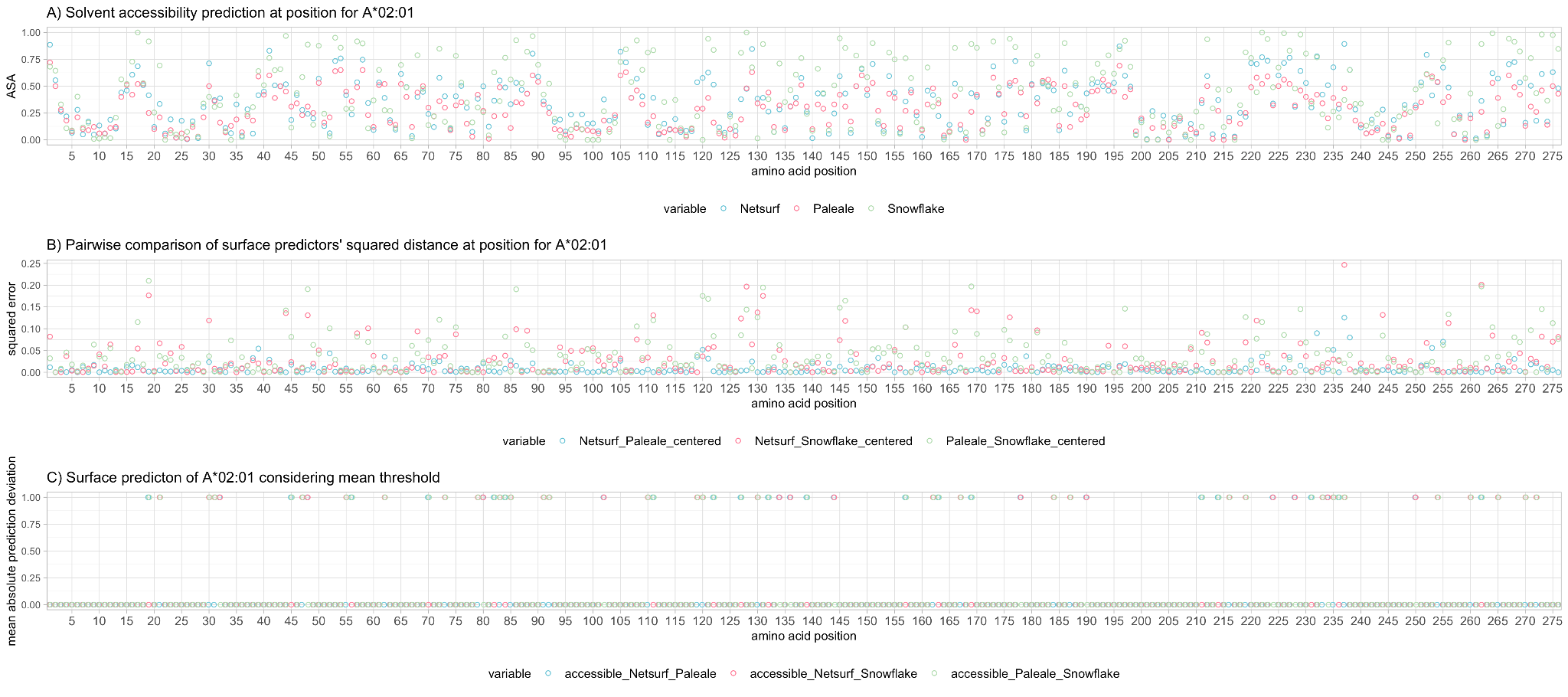


Supplementary Fig 6: (A) Position-specific solvent accessibility prediction for A*02:01. Color depicts the respective predictor. (B) Position-specific pairwise squared distance between the predictors’ centered prediction. Higher values indicate stronger disagreement between the predictors. Color depicts the respective compared predictor pair. (C) Position-specific pairwise disagreement considering the respective predictor’s mean as a threshold for surface accessibility. Residue positions are encoded on the x axis. Scores at zero indicate agreement between predictors, scores at one indicate disagreement between the predictors. Color depicts the respective compared predictor pair.


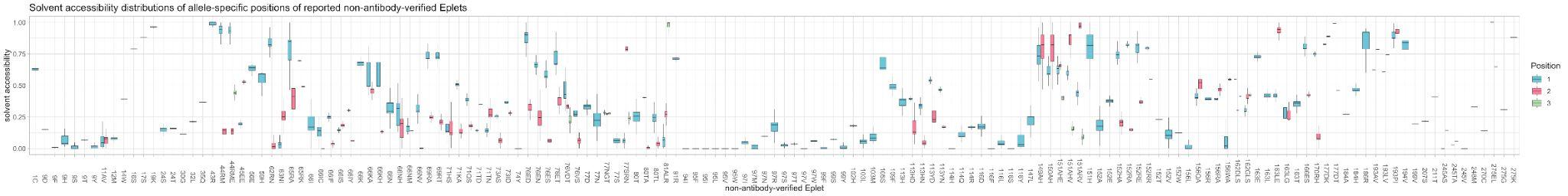


Supplementary Fig 7: Distributions of solvent accessibility of respective non-antibody-verified Eplets positions. Color corresponds to the amino acid position within the Eplet. Boxplots depict the median (horizontal line), first to third quartile (box); the highest and lowest values within 1.5 x IQR (whiskers), respectively.


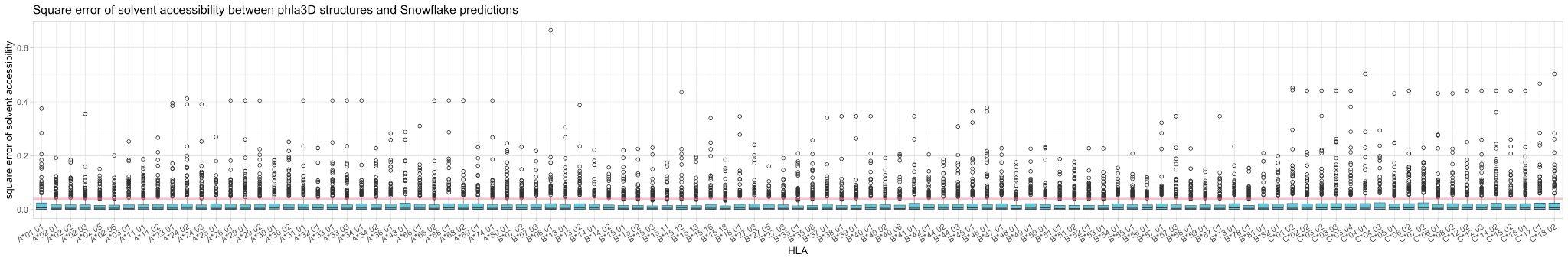


Supplementary Fig 8: Position-wise square error between structures provided by pHLA3D and Snowflake. Boxplots depict the median (horizontal line), first to third quartile (box), outliers outside the 1.5 x IQR (circles), the highest and lowest values within 1.5× IQR (whiskers), respectively. Pink line indicates the a squared error of 0.04 corresponding to a difference of 0.2 in predicted surface area.
